# Advanced soliton structures and elliptic wave patterns in a sixth-order nonlinear Schrödinger equation using improved modified extended tanh function method

**DOI:** 10.1038/s41598-025-30797-6

**Published:** 2025-12-17

**Authors:** Mina M. Fahim, Hamdy M. Ahmed, K. A. Dib, Islam Samir

**Affiliations:** 1 Basic Science Department, Faculty of Engineering, The British University in Egypt, El shorouk, Cairo, Egypt; 2 Mathematics Department, Faculty of Science, Fayoum University, Fayoum, Egypt; 3 Physics and Engineering Mathematics Department, Higher Institute of Engineering, El-shorouk Academy, El- Shorouk City, Cairo, Egypt; 4 Physics and Engineering Mathematics Department, Faculty of Engineering, Ain Shams University, Abbassia, Cairo, Egypt

**Keywords:** Integrable hierarchy systems, Nonlinear dispersive media, Optical systems, Complex pulse modulation, Mathematics and computing, Optics and photonics, Physics

## Abstract

In this work, a sixth–order extension of the nonlinear Schrödinger equation (NLSE) within its integrable hierarchy is investigated to model higher–order nonlinear and dispersive effects relevant to optical fiber systems and nonlinear wave propagation. By employing the Improved Modified Extended Tanh Function Method, a comprehensive family of exact analytical solutions is derived, encompassing bright and dark solitons, singular soliton structures, and singular periodic solutions. In addition, solution families expressed in terms of Jacobi elliptic functions, Weierstrass doubly periodic elliptic functions, and exponential profiles are obtained. The novelty of this study lies in extending the analytical framework of the NLSE hierarchy to its sixth–order integrable form and in uncovering new soliton and elliptic wave structures. The obtained results reveal rich nonlinear dynamics associated with higher–order dispersion and nonlinearity and clarify the transition between periodic and localized behaviors. Two– and three–dimensional graphical simulations further illustrate the spatiotemporal evolution of the derived solutions. Overall, the findings deepen the understanding of advanced nonlinear wave mechanisms and offer potential implications for ultrafast optical and nonlinear waveguide systems.

## Introduction

Nonlinear partial differential equations (NPDEs) play a fundamental role in describing complex dynamical processes in nature and technology. Among the most notable nonlinear wave phenomena are solitons—localized and stable wave packets that preserve their shape and velocity over long propagation distances due to the delicate balance between dispersion and nonlinearity. NPDEs also admit other solution types, such as breathers, periodic waves, and rogue waves, the latter being large-amplitude, localized events that appear transiently and capture extreme dynamics in nonlinear systems^1–3^. A canonical mathematical model that governs such behaviors is the nonlinear Schrödinger equation (NLSE), which appears in diverse physical contexts, including nonlinear optics, plasma physics, hydrodynamics, and quantum field theory. Owing to its integrability, the NLSE admits exact analytical solutions and provides deep insights into nonlinear wave dynamics.

Although the classical NLSE has been widely investigated, it fails to accurately model scenarios in which higher–order dispersive and nonlinear contributions play a significant role, particularly in optical fiber systems supporting ultrashort and high–intensity pulses. Therefore, several integrable higher–order generalizations have been introduced. Prominent examples include the Hirota equation, which incorporates third–order dispersion ^4^, and the Lakshmanan–Porsezian–Daniel (LPD) equation, which accounts for fourth–order effects ^5,6^. These extensions preserve integrability while substantially enhancing the physical fidelity of the governing models.

In many modern photonic and quantum systems, wave packets may evolve on time scales comparable to the carrier frequency, where classical approximations such as the slowly varying envelope approximation cease to be sufficient. In such regimes, ultrashort (sub-picosecond or femtosecond) optical pulses ^7,8^, strongly nonlinear matter waves in Bose–Einstein condensates, and intense Langmuir wave packets in plasma require the inclusion of higher-order dispersive and nonlinear effects ^9^. In fiber optics, for example, third-order dispersion and self-steepening become significant for picosecond pulses, while fourth- and sixth-order dispersion terms become relevant for femtosecond pulses propagating in photonic-crystal fibers, dispersion-engineered fibers, and microstructured waveguides. These higher-order contributions also capture delayed nonlinear responses, Raman-type effects, and ultrafast saturation of the Kerr nonlinearity.

The hierarchical extension of the NLSE, often referred to as the generalized nonlinear Schrödinger hierarchy, has been developed to characterize systems that require successive higher–order corrections while preserving integrability ^10–12^. In this work, we focus on the sixth–order member of this hierarchy, given by

1 $$\begin{aligned} \begin{aligned}&i\psi _x+\frac{1}{2}\psi _{tt}+\psi |\psi |^2-i\alpha _3\left( \psi _{ttt}+6|\psi |^2\psi _{t}\right) +\alpha _{4}\left( \psi _{tttt}+8|\psi |^2 \psi _{tt}+6\psi |\psi |^4+4\psi |\psi _{t}|^2+6\psi _{t}^{2}\psi ^{*}+2\psi ^2\psi ^{*}_{tt}\right) \\&+i \alpha _{5}\left( \psi _{ttttt}+10|\psi |^2\psi _{ttt}+30|\psi |^4\psi _{t}+10\psi \psi _{t}\psi ^{*}_{tt}+10\psi \psi ^{*}_{t}\psi _{tt}+20\psi ^{*}\psi _{t}\psi _{tt}+10\psi ^{2}_{t}\psi ^{*}_{t}\right) +\alpha _{6} \psi _{tttttt} \\&+\alpha _{6}\left[ 60\psi ^{*}|\psi _{t}|^2+50(\psi ^*)^2\psi _{tt}+2\psi ^*_{tttt} \right] \psi ^2+\alpha _{6}\psi \left[ 12\psi ^*\psi _{tttt}+8\psi _t\psi ^*_{ttt}+22|\psi _{tt}|^2 \right] +\alpha _{6}\psi \left[ 18\psi _{ttt}\psi ^*_{t}+70(\psi ^*)^2 \psi _{t}^2 \right] \\&+20\alpha _{6}\psi _t^2\psi _{tt}^*+10\alpha _{6}\psi _t\left[ 5\psi _{tt}\psi ^{*}_{t}+3\psi ^*\psi _{ttt} \right] +20\alpha _{6}\psi ^*\psi ^2_{tt}+10\alpha _{6}\psi ^3\left[ (\psi _t^*)^2+2\psi ^*\psi ^*_{tt} \right] +20\alpha _{6}\psi |\psi |^6=0. \end{aligned} \end{aligned}$$

This equation generalizes well-known integrable NLSE-type models. Specifically, setting $\alpha _5=\alpha _6=0$ yields the LPD equation ^3^, while $\alpha _4=\alpha _5=\alpha _6=0$, recovers the Hirota equation ^10^. Despite the theoretical significance of the sixth-order extension, systematic analytical studies and explicit solution constructions for this model remain scarce in the literature. This motivates the present investigation.

From a physical perspective, the coefficients $\alpha _3, \alpha _4$, $\alpha _5$, and $\alpha _6$ represent progressively higher-order dispersive and nonlinear contributions : $\alpha _3$, and $\alpha _4$ is associated with third, and fourth-order dispersion, nonlinear dispersion, and self-frequency shift effects; $\alpha _5$ governs fifth-order nonlinear dispersion and ultrafast self-steepening interactions; and $\alpha _6$ corresponds to sixth-order dispersion and high-order nonlinear responses, which become significant in the propagation of ultrashort pulses where higher-order spectral broadening and nonlinear refractive index saturation occur. Such effects are particularly relevant in the design of high-power fiber lasers, super continuum generation in photonic-crystal fibers, and the manipulation of matter-wave solitons in condensates subjected to higher-order effective interactions.

Recently, many researchers have been actively modifying and applying various ansatz-based methods and analytical techniques to obtain soliton solutions. Among the commonly used methods are the Fan sub-equation method ^13^, the extended simplest equation method^14^, the new extended auxiliary equation method ^15,16^, the solitary wave ansatz method ^17^, the Sardar sub-equation method ^18^, the modified extended direct algebraic method ^19–21^, the improved modified extended tanh function method ^22–24^, and the Jacobi elliptic function method ^25^, as well as the F-expansion method ^26^. In addition, other effective approaches such as the sine-Gordon method ^27^, the Hirota bilinear method ^28–30^, and the exp($\phi (\zeta )$)-function method ^31^ have also been employed to derive analytical soliton solutions.

Several researchers have investigated the stability of soliton solutions using analytical and numerical methods to better understand their dynamical behavior ^32,33^. These studies provide insight into the robustness and persistence of solitons under perturbations in nonlinear systems.

**Motivation and research gap.** Higher–order NLSE hierarchies are essential for understanding ultrashort pulse propagation and strong nonlinear wave interactions. However, analytical results for the sixth–order NLSE member remain limited. In particular, explicit soliton and periodic solutions, along with their physical characteristics, have not been sufficiently reported. Elucidating the structure of such higher–order solitary waves provides deeper insight into pulse–shaping mechanisms and nonlinear dispersion balances in advanced optical and quantum systems.

**Objective.** The main objective of this work is to derive exact closed-form solutions for the sixth-order NLSE hierarchy and analyze the corresponding nonlinear wave structures.

**Method and contributions.** To achieve this, we utilize the improved modified extended tanh function method, a powerful symbolic technique for solving nonlinear evolution equations. The major contributions are summarized as follows:

- Analytical investigation of the sixth-order NLSE hierarchy and its physical relevance.
- Construction of new exact solutions, including bright, dark, and singular solitons.
- Derivation of additional periodic solutions expressed in terms of Jacobi and Weierstrass elliptic functions.
- Numerical visualization of selected solutions in 2D and 3D to illustrate propagation dynamics.

## Proposed technique

We briefly outline the steps involved in the improved modified extended tanh function method (IMETFM) ^22–24^.

First, we assume that the nonlinear partial differential equation can be expressed in terms of $\psi$ and its partial derivatives as follows:

2 $$\begin{aligned} \mathcal {P}(\psi ,\psi _t,\psi _x,\psi _{xt},\psi _{xx},\ldots ) = 0. \end{aligned}$$

To reduce Eq. (2), we assume a traveling wave transformation for the wave envelope

$$\psi (x,t)=\psi (\zeta ),$$

where $\zeta = x - \nu t$ with $\nu \ne 0$, which converts the function $\psi$ into a function of a single-variable form. Substituting this transformation into Eq. (2) yields the following ordinary differential equation:

3 $$\begin{aligned} \mathcal {P}(\psi ,\psi ^{(1)},\psi ^{(2)},\psi ^{(3)},\psi ^{(4)},\ldots ) = 0. \end{aligned}$$

The next step in applying the IMETFM is to assume that the solution of Eq. (3) has the finite series form

4 $$\begin{aligned} \psi (\zeta ) = \sum _{i=-N}^{N} c_i \phi ^{i}(\zeta ), \end{aligned}$$

where $c_N^2 + c_{-N}^2 \ne 0$, and the function $\phi$ satisfies the extended Riccati equation

5 $$\begin{aligned} \phi ' = \sqrt{d_0 + d_1 \phi + d_2 \phi ^2 + d_3 \phi ^3 + d_4 \phi ^4}. \end{aligned}$$

The integer *N* is determined by applying the homogeneous balance principle, which balances the highest-order derivative term with the highest-order nonlinear term in Eq. (3).

Substituting Eq. (4) into Eq. (3), while taking Eq. (5) into account, results in a polynomial in $\phi$. By equating the coefficients of the obtained polynomial in $\phi$ to zero, we obtain a system of nonlinear algebraic equations involving the parameters of the original equation, the Riccati equation, and the finite series form. This system can be solved using symbolic computation software such as *Mathematica*.

## Methodology and findings

We now apply the improved modified extended tanh function method to Eq.(1). Assuming the wave envelope as:

6 $$\begin{aligned} \psi (x,t) = \mathcal {R}(\zeta )e^{i(\omega t-kx+\theta )}, \end{aligned}$$

where $\zeta = x - \nu t$, and $\nu$, $\theta$, *k*, $\omega$, represent the soliton speed, phase constant, wave number, and soliton frequency, respectively. Inserting the wave envelope assumption in Eq. (6) into Eq. (1), then splitting the obtained equation into its real,and imaginary components, then we get the following:

The real component is given by:

$$\mathcal {R} \left( -2 \alpha _6 \omega ^6 + 2 \alpha _5 \omega ^5 + 2 \alpha _4 \omega ^4 - 2 \alpha _3 \omega ^3 + 2k - \omega ^2\right) + \mathcal {R}^3 \left( 60 \alpha _6 \omega ^4 - 40 \alpha _5 \omega ^3 - 24 \alpha _4 \omega ^2 + 12 \alpha _3 \omega + 2\right) +$$

$$\mathcal {R} \left( \mathcal {R}'\right) ^2 \left( -300 \alpha _6 \nu ^2 \omega ^2 + 100 \alpha _5 \nu ^2 \omega + 20 \alpha _4 \nu ^2\right) + 40 \alpha _6 \mathcal {R}^7 + \mathcal {R}^5 \left( -180 \alpha _6 \omega ^2 + 60 \alpha _5 \omega + 12 \alpha _4\right) +$$

$$280 \alpha _6 \nu ^2 \mathcal {R}^3 \left( \mathcal {R}'\right) ^2 + \mathcal {R}'' \left( 30 \alpha _6 \nu ^2 \omega ^4 - 20 \alpha _5 \nu ^2 \omega ^3 - 12 \alpha _4 \nu ^2 \omega ^2 + 6 \alpha _3 \nu ^2 \omega + \nu ^2\right) +$$

$$84 \alpha _6 \nu ^4 \mathcal {R} \left( \mathcal {R}''\right) ^2 + 112 \alpha _6 \nu ^4 \mathcal {R} \mathcal {R}^{(3)} \mathcal {R}' + 140 \alpha _6 \nu ^4 \left( \mathcal {R}'\right) ^2 \mathcal {R}'' + \mathcal {R}^2 \mathcal {R}'' \left( -300 \alpha _6 \nu ^2 \omega ^2 + 100 \alpha _5 \nu ^2 \omega + 20 \alpha _4 \nu ^2\right) + 140 \alpha _6 \nu ^2 \mathcal {R}^4 \mathcal {R}'' +$$

$$2 \alpha _6 \nu ^6 \mathcal {R}^{(6)} + \mathcal {R}^{(4)} \left( -30 \alpha _6 \nu ^4 \omega ^2 + 10 \alpha _5 \nu ^4 \omega + 2 \alpha _4 \nu ^4\right) + 28 \alpha _6 \nu ^4 \mathcal {R}^2 \mathcal {R}^{(4)} = 0.$$

The imaginary component reads:

$$\left( -12 \alpha _6 \nu \omega ^5 + 10 \alpha _5 \nu \omega ^4 + 8 \alpha _4 \nu \omega ^3 - 6 \alpha _3 \nu \omega ^2 - 2 \nu \omega + 2\right) \mathcal {R}' + \left( 240 \alpha _6 \nu \omega ^3 - 120 \alpha _5 \nu \omega ^2 - 48 \alpha _4 \nu \omega + 12 \alpha _3 \nu \right) \mathcal {R}^2 \mathcal {R}' +$$

$$\left( 20 \alpha _5 \nu ^3 - 120 \alpha _6 \nu ^3 \omega \right) \mathcal {R}'^3 + \left( 80 \alpha _5 \nu ^3 - 480 \alpha _6 \nu ^3 \omega \right) \mathcal {R} \mathcal {R}' \mathcal {R}'' + \left( 60 \alpha _5 \nu - 360 \alpha _6 \nu \omega \right) \mathcal {R}^4 \mathcal {R}' +$$

$$\left( 2 \alpha _5 \nu ^5 - 12 \alpha _6 \nu ^5 \omega \right) \mathcal {R}^{(5)} + \left( 40 \alpha _6 \nu ^3 \omega ^3 - 20 \alpha _5 \nu ^3 \omega ^2 - 8 \alpha _4 \nu ^3 \omega + 2 \alpha _3 \nu ^3\right) \mathcal {R}^{(3)} + \left( 20 \alpha _5 \nu ^3 - 120 \alpha _6 \nu ^3 \omega \right) \mathcal {R}^{(3)} \mathcal {R}^2 = 0.$$

Equating the coefficients of the imaginary parts to zero, we derive the following relationships:

$$\begin{aligned} \left\{ \begin{aligned} \nu&= \frac{1250 \alpha _5^3}{216 \alpha _4^4 + 810 \alpha _3 \alpha _5 \alpha _4^2 - 72 \alpha _4^3 \mathcal {L} - 15 \alpha _5 \alpha _4 \left( 25 \alpha _5 + 14 \alpha _3 \mathcal {L}\right) + 25 \alpha _5^2 \left( 18 \alpha _3^2 + 5 \mathcal {L}\right) }, \\ \omega&= \frac{\mathcal {L} - 3 \alpha _4}{10 \alpha _5}, \\ \alpha _6&= \frac{\alpha _5 (\mathcal {L} + 3 \alpha _4)}{9 \alpha _3}, \end{aligned} \right. \end{aligned}$$

where $$\mathcal {L} = \sqrt{9 \alpha _4^2 + 15 \alpha _3 \alpha _5}$$.

Applying the balancing principle by balancing the highest order derivative term $\mathcal {R}^{(6)}$ with the highest nonlinear term $\mathcal {R}^7$ in the real part, we obtain that $N = 1$. Consequently, we express $\mathcal {R}(\zeta )$ as:

7 $$\begin{aligned} \mathcal {R}(\zeta ) = c_0 + c_1 \phi + c_{-1} \phi ^{-1}. \end{aligned}$$

Substituting Eq. (7) into the real part and considering the extended Riccati equation in Eq. (5), we obtain a polynomial in $\phi$. By equating the coefficients of this polynomial to zero, we derive different algebraic systems based on the values of $d_0, d_1, d_2, d_3, d_4$, which are then solved using Mathematica.

### Localized solitons

When $\{d_0=d_1=d_3=0\}$, we attain the following result.

$$\begin{aligned} \left\{ \begin{aligned} c_1&= \sqrt{-d_4}\,\nu , \qquad c_0 = c_{-1} = 0, \\ k&= \tfrac{1}{2}\Big [ \omega ^{2} - 2\nu ^{6} d_{2}^{3}\alpha _{6} + 2\omega ^{3}\!\big (\alpha _{3} - \omega (\alpha _{4} + \omega \alpha _{5}) + \omega ^{3}\alpha _{6}\big ) \\&\quad - \nu ^{2} d_{2}\!\big (1 + 6\omega \alpha _{3} - 4\omega ^{2}(3\alpha _{4} + 5\omega \alpha _{5}) + 30\omega ^{4}\alpha _{6}\big ) - 2\nu ^{4} d_{2}^{2}\!\big (\alpha _{4} + 5\omega (\alpha _{5} - 3\omega \alpha _{6})\big ) \Big ]. \end{aligned} \right. \end{aligned}$$

Then, the preceding solution set admits the following bright soliton.

8 $$\begin{aligned} \psi (x,t)= \frac{1250 \alpha _5^3 \sqrt{d_2} \text {sech}\left( \sqrt{d_2} \zeta \right) \times e^{i (\theta -k x+t \omega )}}{216 \alpha _4^4-72 \mathcal {L} \alpha _4^3+810 \alpha _3 \alpha _5 \alpha _4^2-15 \alpha _5 \left( 14 \mathcal {L} \alpha _3+25 \alpha _5\right) \alpha _4+25 \left( 18 \alpha _3^2+5 \mathcal {L}\right) \alpha _5^2}, \end{aligned}$$

When $\{ d_1=d_3=0$, $d_0=\frac{d_2^2}{4 d_4}\}$, we attain the following result.

$$\begin{aligned} \left\{ \begin{aligned} c_1&= \sqrt{-d_4}\,\nu , \qquad c_0 = c_{-1} = 0, \\ k&= \tfrac{1}{2}\Big ( -5\nu ^6 d_2^3 \alpha _6 - 3\nu ^4 d_2^2 \big (\alpha _4 + 5\omega (\alpha _5 - 3\omega \alpha _6)\big ) \\&\quad + \omega ^2 \big (1 + 2\omega (\alpha _3 - \omega (\alpha _4 + \omega \alpha _5) + \omega ^3\alpha _6)\big ) + \nu ^2 d_2 \big (-1 - 6\omega \alpha _3 + 2\omega ^2(6\alpha _4 + 5\omega (2\alpha _5 - 3\omega \alpha _6))\big ) \Big ). \end{aligned} \right. \end{aligned}$$

Then, We get the following dark soliton.

9 $$\begin{aligned} \psi (x,t)= \frac{625 \sqrt{2} \alpha _5^3 \sqrt{d_2} \tanh \left( \frac{\sqrt{-d_2} \zeta }{\sqrt{2}}\right) \times e^{i (\theta -k x+t \omega )}}{216 \alpha _4^4-72 \mathcal {L} \alpha _4^3+810 \alpha _3 \alpha _5 \alpha _4^2-15 \alpha _5 \left( 14 \mathcal {L} \alpha _3+25 \alpha _5\right) \alpha _4+25 \left( 18 \alpha _3^2+5 \mathcal {L}\right) \alpha _5^2}, \end{aligned}$$

When $\{d_0=d_1=0,\;\;d_2= \frac{d_3^2}{4 d_4}\}$, we get the following result.

$$\begin{aligned} \left\{ \begin{aligned} c_0&= \frac{i d_3 \nu }{4 \sqrt{d_4}}, \quad c_1 = i \sqrt{d_4}\, \nu , \quad c_{-1} = 0, \\ k&= \frac{1}{1024 d_4^3} \Big [ 5 \alpha _6 d_3^6 \nu ^6 - 24 d_4 d_3^4 \nu ^4 \!\left( 5\omega (\alpha _5 - 3 \alpha _6 \omega ) + \alpha _4 \right) \\&\quad + 64 d_4^2 d_3^2 \nu ^2 \!\left( 30 \alpha _6 \omega ^4 - 4 \omega ^2 (5 \alpha _5 \omega + 3 \alpha _4) + 6 \alpha _3 \omega + 1 \right) \\&\quad + 512 d_4^3 \omega ^2 \!\left( 2 \omega (\alpha _6 \omega ^3 - \omega (\alpha _5 \omega + \alpha _4) + \alpha _3) + 1 \right) \Big ]. \end{aligned} \right. \end{aligned}$$

Then, the preceding result yields the following dark soliton.

10 $$\begin{aligned} \psi (x,t)= -\frac{625 i \alpha _5^3 \left( 2 \sqrt{d_2 d_4} \left( \tanh \left( \frac{\sqrt{d_2} \zeta }{2}\right) +1\right) +d_3\right) \times e^{i (\theta -k x+t \omega )}}{2 \sqrt{d_4} \left( -216 \alpha _4^4-810 \alpha _3 \alpha _5 \alpha _4^2+72 \alpha _4^3 \mathcal {L}+15 \alpha _5 \alpha _4 \left( 25 \alpha _5+14 \alpha _3 \mathcal {L}\right) -25 \alpha _5^2 \left( 18 \alpha _3^2+5 \mathcal {L}\right) \right) }. \end{aligned}$$

### Singular periodic solutions and singular solitons

When $\{d_3=d_4=d_1=0\}$, we get the following solution set :

$$\begin{aligned} \left\{ \begin{aligned} c_0&= c_1 = 0, \quad c_{-1} = \sqrt{-d_0}\,\nu , \\ k&= \frac{1}{2}\Big [ 2\omega ^3\!\left( \alpha _6 \omega ^3 - \omega (\alpha _5 \omega + \alpha _4) + \alpha _3\right) - 2\alpha _6 d_2^3 \nu ^6 - 2 d_2^2 \nu ^4 \!\left( 5\omega (\alpha _5 - 3\alpha _6 \omega ) + \alpha _4\right) \\&\quad - d_2 \nu ^2 \!\left( 30 \alpha _6 \omega ^4 - 4 \omega ^2 (5 \alpha _5 \omega + 3 \alpha _4) + 6 \alpha _3 \omega + 1\right) + \omega ^2 \Big ]. \end{aligned} \right. \end{aligned}$$

Consequently, we obtain the following singular periodic solution and singular soliton, respectively.

11 $$\begin{aligned} \psi (x,t)= \frac{1250 \alpha _5^3 \sqrt{d_2} \csc \left( \sqrt{-d_2} \zeta \right) \times e^{i (\theta -k x+t \omega )} }{216 \alpha _4^4+810 \alpha _3 \alpha _5 \alpha _4^2-72 \alpha _4^3 \mathcal {L}-15 \alpha _5 \alpha _4 \left( 25 \alpha _5+14 \alpha _3 \mathcal {L}\right) +25 \alpha _5^2 \left( 18 \alpha _3^2+5 \mathcal {L}\right) }, \end{aligned}$$

12 $$\begin{aligned} \psi (x,t)= \frac{1250 \alpha _5^3 \sqrt{-d_2} \text {csch}\left( \sqrt{-d_2} \zeta \right) \times e^{i (\theta -k x+t \omega )} }{216 \alpha _4^4+810 \alpha _3 \alpha _5 \alpha _4^2-72 \alpha _4^3 \mathcal {L}-15 \alpha _5 \alpha _4 \left( 25 \alpha _5+14 \alpha _3 \mathcal {L}\right) +25 \alpha _5^2 \left( 18 \alpha _3^2+5\mathcal {L}\right) }. \end{aligned}$$

When $\{ d_1=d_3=0$, $d_0=\frac{d_2^2}{4 d_4}\}$, we obtain the following results.

#### Result 1

$$\begin{aligned} \left\{ \begin{aligned} c_{-1}&= \frac{i d_2 \nu }{2\sqrt{d_4}}, \quad c_0 = c_1 = 0, \\ k&= \tfrac{1}{2}\Big [ -5\nu ^6 d_2^3 \alpha _6 - 3\nu ^4 d_2^2(\alpha _4 + 5\omega (\alpha _5 - 3\omega \alpha _6)) + \omega ^2\!\big (1 + 2\omega (\alpha _3 - \omega (\alpha _4 + \omega \alpha _5) + \omega ^3\alpha _6)\big ) \\&\quad + \nu ^2 d_2\!\big (-1 - 6\omega \alpha _3 + 2\omega ^2(6\alpha _4 + 5\omega (2\alpha _5 - 3\omega \alpha _6))\big ) \Big ]. \end{aligned} \right. \end{aligned}$$

Consequently, the previous result admits the following singular periodic solution, and singular soliton, respectively.

13 $$\begin{aligned} \psi (x,t)= \frac{625 i \sqrt{2} \alpha _5^3 d_2 \cot \left( \frac{\sqrt{d_2} \zeta }{\sqrt{2}}\right) \times e^{i (\theta -k x+t \omega )}}{\sqrt{d_2} \left( 216 \alpha _4^4+810 \alpha _3 \alpha _5 \alpha _4^2-72 \alpha _4^3 \mathcal {L}-15 \alpha _5 \alpha _4 \left( 25 \alpha _5+14 \alpha _3 \mathcal {L}\right) +25 \alpha _5^2 \left( 18 \alpha _3^2+5 \mathcal {L}\right) \right) }, \end{aligned}$$

14 $$\begin{aligned} \psi (x,t)= \frac{625 \sqrt{2} \alpha _5^3 d_2 \coth \left( \frac{\sqrt{-d_2} \zeta }{\sqrt{2}}\right) \times e^{i (\theta -k x+t \omega )}}{\sqrt{d_2} \left( 216 \alpha _4^4+810 \alpha _3 \alpha _5 \alpha _4^2-72 \alpha _4^3 \mathcal {L}-15 \alpha _5 \alpha _4 \left( 25 \alpha _5+14 \alpha _3 \mathcal {L}\right) +25 \alpha _5^2 \left( 18 \alpha _3^2+5 \mathcal {L}\right) \right) }. \end{aligned}$$

#### Result 2

$$\begin{aligned} \left\{ \begin{aligned} c_0&= 0, \quad c_1 = i\sqrt{d_4}\,\nu , \quad c_{-1} = \frac{i d_2 \nu }{2\sqrt{d_4}}, \\ k&= \tfrac{1}{2}\Big [ -5\nu ^6 d_2^3 \alpha _6 - 3\nu ^4 d_2^2\!\big (\alpha _4 + 5\omega (\alpha _5 - 3\omega \alpha _6)\big ) \\&\quad + \omega ^2\!\big (1 + 2\omega (\alpha _3 - \omega (\alpha _4 + \omega \alpha _5) + \omega ^3\alpha _6)\big ) + \nu ^2 d_2\!\big (-1 - 6\omega \alpha _3 + 2\omega ^2(6\alpha _4 + 5\omega (2\alpha _5 - 3\omega \alpha _6))\big ) \Big ]. \end{aligned} \right. \end{aligned}$$

Then, we associate with the preceding solution set, the following singular soliton, and singular periodic solution, respectively.

15 $$\begin{aligned} \psi (x,t)= \frac{1250 \sqrt{2} \alpha _5^3 \sqrt{d_2} \text {csch}\left( \sqrt{2} \sqrt{-d_2} \zeta \right) \times e^{i (\theta -k x+t \omega )}}{-216 \alpha _4^4-810 \alpha _3 \alpha _5 \alpha _4^2+72 \alpha _4^3 \mathcal {L}+15 \alpha _5 \alpha _4 \left( 25 \alpha _5+14 \alpha _3 \mathcal {L}\right) -25 \alpha _5^2 \left( 18 \alpha _3^2+5 \mathcal {L}\right) }, \end{aligned}$$

16 $$\begin{aligned} \psi (x,t)= \frac{1250 i \sqrt{2} \alpha _5^3 \sqrt{d_2} \csc \left( \sqrt{2} \sqrt{d_2} \zeta \right) \times e^{i (\theta -k x+t \omega )}}{216 \alpha _4^4+810 \alpha _3 \alpha _5 \alpha _4^2-72 \alpha _4^3 \mathcal {L}-15 \alpha _5 \alpha _4 \left( 25 \alpha _5+14 \alpha _3 \mathcal {L}\right) +25 \alpha _5^2 \left( 18 \alpha _3^2+5 \mathcal {L}\right) }. \end{aligned}$$

When $\{d_0=d_1=d_3=0\}$, we attain the following result.

$$\begin{aligned} \left\{ \begin{aligned} c_1&= \sqrt{-d_4}\,\nu , \qquad c_0 = c_{-1} = 0, \\ k&= \tfrac{1}{2}\Big [ \omega ^{2} - 2\nu ^{6} d_{2}^{3}\alpha _{6} + 2\omega ^{3}\!\big (\alpha _{3} - \omega (\alpha _{4} + \omega \alpha _{5}) + \omega ^{3}\alpha _{6}\big ) \\&\quad - \nu ^{2} d_{2}\!\big (1 + 6\omega \alpha _{3} - 4\omega ^{2}(3\alpha _{4} + 5\omega \alpha _{5}) + 30\omega ^{4}\alpha _{6}\big ) - 2\nu ^{4} d_{2}^{2}\!\big (\alpha _{4} + 5\omega (\alpha _{5} - 3\omega \alpha _{6})\big ) \Big ]. \end{aligned} \right. \end{aligned}$$

Then, the following singular periodic solution is obtained

17 $$\begin{aligned} \psi (x,t)= \frac{1250 \alpha _5^3 \sqrt{d_2} \sec \left( \sqrt{-d_2} \zeta \right) \times e^{i (\theta -k x+t \omega )}}{216 \alpha _4^4+810 \alpha _3 \alpha _5 \alpha _4^2-72 \alpha _4^3 \mathcal {L}-15 \alpha _5 \alpha _4 \left( 25 \alpha _5+14 \alpha _3 \mathcal {L}\right) +25 \alpha _5^2 \left( 18 \alpha _3^2+5 \mathcal {L}\right) }. \end{aligned}$$

When $\{ d_1=d_3=0$, $d_0=\frac{d_2^2}{4 d_4}\}$, the following result is obtained.

$$\begin{aligned} \left\{ \begin{aligned} c_1&= \sqrt{-d_4}\,\nu , \qquad c_0 = c_{-1} = 0, \\ k&= \tfrac{1}{2}\Big ( -5\nu ^6 d_2^3 \alpha _6 - 3\nu ^4 d_2^2 \big (\alpha _4 + 5\omega (\alpha _5 - 3\omega \alpha _6)\big ) \\&\quad + \omega ^2 \big (1 + 2\omega (\alpha _3 - \omega (\alpha _4 + \omega \alpha _5) + \omega ^3\alpha _6)\big ) + \nu ^2 d_2 \big (-1 - 6\omega \alpha _3 + 2\omega ^2(6\alpha _4 + 5\omega (2\alpha _5 - 3\omega \alpha _6))\big ) \Big ). \end{aligned} \right. \end{aligned}$$

Then, the latter admits the following singular periodic solution.

18 $$\begin{aligned} \psi (x,t)= \frac{625 \sqrt{2} \alpha _5^3 \sqrt{-d_2} \tan \left( \frac{\sqrt{d_2} \zeta }{\sqrt{2}}\right) \times e^{i (\theta -k x+t \omega )}}{216 \alpha _4^4-72 \mathcal {L} \alpha _4^3+810 \alpha _3 \alpha _5 \alpha _4^2-15 \alpha _5 \left( 14 \mathcal {L} \alpha _3+25 \alpha _5\right) \alpha _4+25 \left( 18 \alpha _3^2+5 \mathcal {L}\right) \alpha _5^2}. \end{aligned}$$

### Jacobi elliptic functions and weierstrass solution

When $\{ d_1=d_3=0$, $d_0=\frac{d_2^2 m^2 \left( 1-m^2\right) }{d_4 \left( 2 m^2-1\right) ^2}\}$, we get the following results.

#### Result 1

$$\begin{aligned} \left\{ \begin{aligned} c_1&= \sqrt{-d_4}\,\nu , \quad c_0 = c_{-1} = 0, \\ k&= \omega ^3\!\left( \alpha _6 \omega ^3 - \omega (\alpha _5 \omega + \alpha _4) + \alpha _3\right) - \frac{1}{2} d_2 \nu ^2\!\left( 30 \alpha _6 \omega ^4 - 4 \omega ^2 (5 \alpha _5 \omega + 3 \alpha _4) + 6 \alpha _3 \omega + 1\right) \\&\quad + \frac{\alpha _6 d_2^3 (2 m^4 - 2 m^2 - 1) \nu ^6}{(1 - 2 m^2)^2} - \frac{d_2^2 (2 m^4 - 2 m^2 + 1) \nu ^4 \left[ 5 \omega (\alpha _5 - 3 \alpha _6 \omega ) + \alpha _4\right] }{(1 - 2 m^2)^2} + \frac{\omega ^2}{2}, \end{aligned} \right. \end{aligned}$$

which admits the following Jacobi elliptic function solution.

19 $$\begin{aligned} \psi (x,t)= \frac{1250 \alpha _5^3 \sqrt{\frac{d_2 m^2}{2 m^2-1}} \text {cn}\left( \left. \zeta \sqrt{\frac{d_2}{2 m^2-1}}\right| m\right) \times e^{i (\theta -k x+t \omega )}}{216 \alpha _4^4+810 \alpha _3 \alpha _5 \alpha _4^2-72 \alpha _4^3 \mathcal {L}-15 \alpha _5 \alpha _4 \left( 25 \alpha _5+14 \alpha _3 \mathcal {L}\right) +25 \alpha _5^2 \left( 18 \alpha _3^2+5 \mathcal {L}\right) }. \end{aligned}$$

#### Result 2

$$\begin{aligned} \left\{ \begin{aligned} c_1&= c_0 = 0, \quad c_{-1} = \frac{i d_2 m \sqrt{m^2-1} \,\nu }{\sqrt{-4 d_4 m^4 + 4 d_4 m^2 - d_4}}, \\ k&= \omega ^3\!\left( \alpha _6 \omega ^3 - \omega (\alpha _5 \omega + \alpha _4) + \alpha _3\right) - \frac{1}{2} d_2 \nu ^2\!\left( 30 \alpha _6 \omega ^4 - 4 \omega ^2 (5 \alpha _5 \omega + 3 \alpha _4) + 6 \alpha _3 \omega + 1\right) \\&\quad + \frac{\alpha _6 d_2^3 (2 m^4 - 2 m^2 - 1) \nu ^6}{(1 - 2 m^2)^2} - \frac{d_2^2 (2 m^4 - 2 m^2 + 1) \nu ^4 \left[ 5 \omega (\alpha _5 - 3 \alpha _6 \omega ) + \alpha _4\right] }{(1 - 2 m^2)^2} + \frac{\omega ^2}{2}, \end{aligned} \right. \end{aligned}$$

which admits another Jacobi elliptic solution.

20 $$\begin{aligned} \psi (x,t)= \frac{1250 \alpha _5^3 d_2 \sqrt{1-m^2} \text {cn}\left( \left. \zeta \sqrt{\frac{d_2}{2 m^2-1}}\right| m\right) ^{-1} \times e^{i (\theta -k x+t \omega )}}{\sqrt{d_2 \left( 2 m^2-1\right) } \left( 216 \alpha _4^4+810 \alpha _3 \alpha _5 \alpha _4^2-72 \alpha _4^3 \mathcal {L}-15 \alpha _5 \alpha _4 \left( 25 \alpha _5+14 \alpha _3 \mathcal {L}\right) +25 \alpha _5^2 \left( 18 \alpha _3^2+5 \mathcal {L}\right) \right) }. \end{aligned}$$

When $\{ d_1=d_3=0$, $d_0=\frac{d_2^2 \left( 1-m^2\right) }{d_4 \left( 2-m^2\right) ^2}\}$, we get the following results.

#### Result 1

$$\begin{aligned} \left\{ \begin{aligned} c_0&= c_{-1} = 0, \quad c_1 = \sqrt{-d_4}\,\nu , \\ k&= \frac{1}{2}\Big [ -\frac{2(10 - 10 m^2 + m^4)\,\nu ^6 d_2^3 \alpha _6}{(-2 + m^2)^2} - \frac{2(6 - 6 m^2 + m^4)\,\nu ^4 d_2^2 (\alpha _4 + 5\omega (\alpha _5 - 3\omega \alpha _6))}{(-2 + m^2)^2} \\&\quad + \omega ^2 \big (1 + 2\omega (\alpha _3 - \omega (\alpha _4 + \omega \alpha _5) + \omega ^3\alpha _6)\big ) + \nu ^2 d_2 \big (-1 - 6\omega \alpha _3 + 2\omega ^2(6\alpha _4 + 5\omega (2\alpha _5 - 3\omega \alpha _6))\big ) \Big ]. \end{aligned} \right. \end{aligned}$$

Then, we obtain the following Jacobi elliptic function solution.

21 $$\begin{aligned} \psi (x,t)= \frac{1250 \alpha _5^3 \sqrt{\frac{m^2}{2-m^2}} \text {dn}\left( \left. \zeta \sqrt{\frac{d_2}{2-m^2}}\right| m\right) \times e^{i (\theta -k x+t \omega )}}{216 \alpha _4^4+810 \alpha _3 \alpha _5 \alpha _4^2-72 \alpha _4^3 \mathcal {L}-15 \alpha _5 \alpha _4 \left( 25 \alpha _5+14 \alpha _3 \mathcal {L}\right) +25 \alpha _5^2 \left( 18 \alpha _3^2+5 \mathcal {L}\right) }. \end{aligned}$$

#### Result 2

$$\begin{aligned} \left\{ \begin{aligned} c_1&= c_0 = 0, \quad c_{-1} = \frac{i d_2 m \sqrt{m^2-1}\,\nu }{\sqrt{-4 d_4 m^4 + 4 d_4 m^2 - d_4}}, \\ k&= \frac{1}{2}\Big [ -\frac{2(10 - 10 m^2 + m^4)\,\nu ^6 d_2^3 \alpha _6}{(-2 + m^2)^2} - \frac{2(6 - 6 m^2 + m^4)\,\nu ^4 d_2^2 (\alpha _4 + 5\omega (\alpha _5 - 3\omega \alpha _6))}{(-2 + m^2)^2} \\&\quad + \omega ^2 \big (1 + 2\omega (\alpha _3 - \omega (\alpha _4 + \omega \alpha _5) + \omega ^3\alpha _6)\big ) + \nu ^2 d_2 \big (-1 - 6\omega \alpha _3 + 2\omega ^2(6\alpha _4 + 5\omega (2\alpha _5 - 3\omega \alpha _6))\big ) \Big ], \end{aligned} \right. \end{aligned}$$

which admits another Jacobi elliptic function solution.

22 $$\begin{aligned} \psi (x,t)= \frac{1250 \alpha _5^3 d_2 \sqrt{1-m^2}\text {dn}\left( \left. \zeta \sqrt{\frac{d_2}{2-m^2}}\right| m\right) ^{-1}\times e^{i (\theta -k x+t \omega )}}{m \sqrt{2-m^2} \left( 216 \alpha _4^4+810 \alpha _3 \alpha _5 \alpha _4^2-72 \alpha _4^3 \mathcal {L}-15 \alpha _5 \alpha _4 \left( 25 \alpha _5+14 \alpha _3 \mathcal {L}\right) +25 \alpha _5^2 \left( 18 \alpha _3^2+5 \mathcal {L}\right) \right) }. \end{aligned}$$

When $\{ d_1=d_3=0$, $d_0=\frac{d_2^2 m^2}{d_4 \left( m^2+1\right) ^2}\}$, we get the following results.

#### Result 1

$$\begin{aligned} \left\{ \begin{aligned} c_0&= c_{-1} = 0, \quad c_1 = \sqrt{-d_4}\,\nu , \\ k&= \frac{1}{2}\Big [ -\frac{2(1 + 8 m^2 + m^4)\,\nu ^6 d_2^3 \alpha _6}{(1 + m^2)^2} - \frac{2(1 + 4 m^2 + m^4)\,\nu ^4 d_2^2 (\alpha _4 + 5\omega (\alpha _5 - 3\omega \alpha _6))}{(1 + m^2)^2} \\&\quad + \omega ^2 \big (1 + 2\omega (\alpha _3 - \omega (\alpha _4 + \omega \alpha _5) + \omega ^3\alpha _6)\big ) + \nu ^2 d_2 \big (-1 - 6\omega \alpha _3 + 2\omega ^2(6\alpha _4 + 5\omega (2\alpha _5 - 3\omega \alpha _6))\big ) \Big ], \end{aligned} \right. \end{aligned}$$

which reads the following Jacobi elliptic solution.

23 $$\begin{aligned} \psi (x,t)= \frac{1250 \alpha _5^3 \sqrt{\frac{d_2 m^2}{m^2+1}} \text {sn}\left( \left. \zeta \sqrt{-\frac{d_2}{m^2+1}}\right| m\right) \times e^{i (\theta -k x+t \omega )}}{216 \alpha _4^4+810 \alpha _3 \alpha _5 \alpha _4^2-72 \alpha _4^3 \mathcal {L}-15 \alpha _5 \alpha _4 \left( 25 \alpha _5+14 \alpha _3 \mathcal {L}\right) +25 \alpha _5^2 \left( 18 \alpha _3^2+5 \mathcal {L}\right) }. \end{aligned}$$

#### Result 2

$$\begin{aligned} \left\{ \begin{aligned} c_0&= c_1 = 0, \quad c_{-1} = \frac{d_2 m \nu }{\sqrt{-d_4 m^4 - 2 d_4 m^2 - d_4}}, \\ k&= \frac{1}{2}\Big [ -\frac{2(1 + 8 m^2 + m^4)\,\nu ^6 d_2^3 \alpha _6}{(1 + m^2)^2} - \frac{2(1 + 4 m^2 + m^4)\,\nu ^4 d_2^2 (\alpha _4 + 5\omega (\alpha _5 - 3\omega \alpha _6))}{(1 + m^2)^2} \\&\quad + \omega ^2 \big (1 + 2\omega (\alpha _3 - \omega (\alpha _4 + \omega \alpha _5) + \omega ^3\alpha _6)\big ) + \nu ^2 d_2 \big (-1 - 6\omega \alpha _3 + 2\omega ^2(6\alpha _4 + 5\omega (2\alpha _5 - 3\omega \alpha _6))\big ) \Big ], \end{aligned} \right. \end{aligned}$$

which derive the following Jacobi elliptic solution.

24 $$\begin{aligned} \psi (x,t)= \frac{1250 \alpha _5^3 d_2\text {sn}\left( \left. \zeta \sqrt{-\frac{d_2}{m^2+1}}\right| m\right) ^{-1}\times e^{i (\theta -k x+t \omega )}}{\left( \sqrt{\frac{d_2 \left( m^2+1\right) }{d_4}} \left( 216 \alpha _4^4+810 \alpha _3 \alpha _5 \alpha _4^2-72 \alpha _4^3 \mathcal {L}-15 \alpha _5 \alpha _4 \left( 25 \alpha _5+14 \alpha _3 \mathcal {L}\right) +25 \alpha _5^2 \left( 18 \alpha _3^2+5 \mathcal {L}\right) \right) \right) }. \end{aligned}$$

When $\{d_2=d_4=0\}$, the following result is revealed.

$$\begin{aligned} \left\{ \begin{aligned} c_{-1}&= \sqrt{-d_0}\, \nu , \quad c_1 = 0, \\ d_3&= \frac{8 c_0^3}{\sqrt{-d_0}\, \nu ^3}, \quad d_1 = -\frac{4 c_0 \sqrt{-d_0}}{\nu }, \\ k&= \omega ^3\!\left( \alpha _6 \omega ^3 - \omega (\alpha _5 \omega + \alpha _4) + \alpha _3\right) - 3 c_0^2\!\left( 30 \alpha _6 \omega ^4 - 4 \omega ^2 (5 \alpha _5 \omega + 3 \alpha _4) + 6 \alpha _3 \omega + 1\right) \\&\quad - 46 c_0^4\!\left( 5 \omega (\alpha _5 - 3 \alpha _6 \omega ) + \alpha _4\right) - 396 \alpha _6 c_0^6 + \frac{\omega ^2}{2}. \end{aligned} \right. \end{aligned}$$

Then, we attain the following Weierstrass elliptic function solution.

25 $$\begin{aligned} \psi (x,t)= \left( c_0+\frac{1250 i \alpha _5^3 \sqrt{d_0}\wp \left( \zeta ;-\frac{4 d_1}{d_3},-\frac{4 d_0}{d_3}\right) ^{-1}}{216 \alpha _4^4+810 \alpha _3 \alpha _5 \alpha _4^2-72 \alpha _4^3 \mathcal {L}-15 \alpha _5 \alpha _4 \left( 25 \alpha _5+14 \alpha _3 \mathcal {L}\right) +25 \alpha _5^2 \left( 18 \alpha _3^2+5 \mathcal {L}\right) } \right) \times e^{i (\theta -k x+t \omega )} . \end{aligned}$$

### Exponential solution

When $\{d_3=d_4=0, d_0=\frac{d_1^2}{4 d_2}\}$, the following result is obtained.

$$\begin{aligned} \left\{ \begin{aligned} c_1&= 0, \quad c_0 = \frac{1}{2} i \sqrt{d_2}\, \nu , \quad c_{-1} = \frac{i d_1 \nu }{2 \sqrt{d_2}}, \\ k&= \frac{1}{16}\Big [ 8 \omega ^2 \!\left( 2\omega (\alpha _6 \omega ^3 - \omega (\alpha _5 \omega + \alpha _4) + \alpha _3) + 1 \right) + 5 \alpha _6 d_2^3 \nu ^6 \\&\quad - 6 d_2^2 \nu ^4 \!\left( 5\omega (\alpha _5 - 3 \alpha _6 \omega ) + \alpha _4 \right) + 4 d_2 \nu ^2 \!\left( 30 \alpha _6 \omega ^4 - 4 \omega ^2 (5 \alpha _5 \omega + 3 \alpha _4) + 6 \alpha _3 \omega + 1 \right) \Big ]. \end{aligned} \right. \end{aligned}$$

Then, We obtain the following exponential solution.

26 $$\begin{aligned} \psi (x,t)= \frac{625 i \alpha _5^3 \sqrt{d_2} \left( 2 d_2 e^{\sqrt{d_2} \zeta }+d_1\right) \left( d_1-2 d_2 e^{\sqrt{d_2} \zeta }\right) ^{-1}\times e^{i (\theta -k x+t \omega )}}{ \left( 216 \alpha _4^4+810 \alpha _3 \alpha _5 \alpha _4^2-72 \alpha _4^3 \mathcal {L}-15 \alpha _5 \alpha _4 \left( 25 \alpha _5+14 \alpha _3 \mathcal {L}\right) +25 \alpha _5^2 \left( 18 \alpha _3^2+5 \mathcal {L}\right) \right) }. \end{aligned}$$

## Results and discussion

This section presents the dynamical behavior of the obtained solutions for the sixth–order integrable NLSE. Five classes of nonlinear wave structures are examined: bright solitons, dark solitons, singular solitons, singular periodic solutions, and Jacobi elliptic solutions. In the case of the Jacobi elliptic solution, the relation and transition between the periodic and localized (soliton) structures are demonstrated. For each case, both 2D and 3D plots illustrate the evolution of the wave amplitude over space and time, confirming the analytical results.

### Bright soliton dynamics

**Fig. 1:**
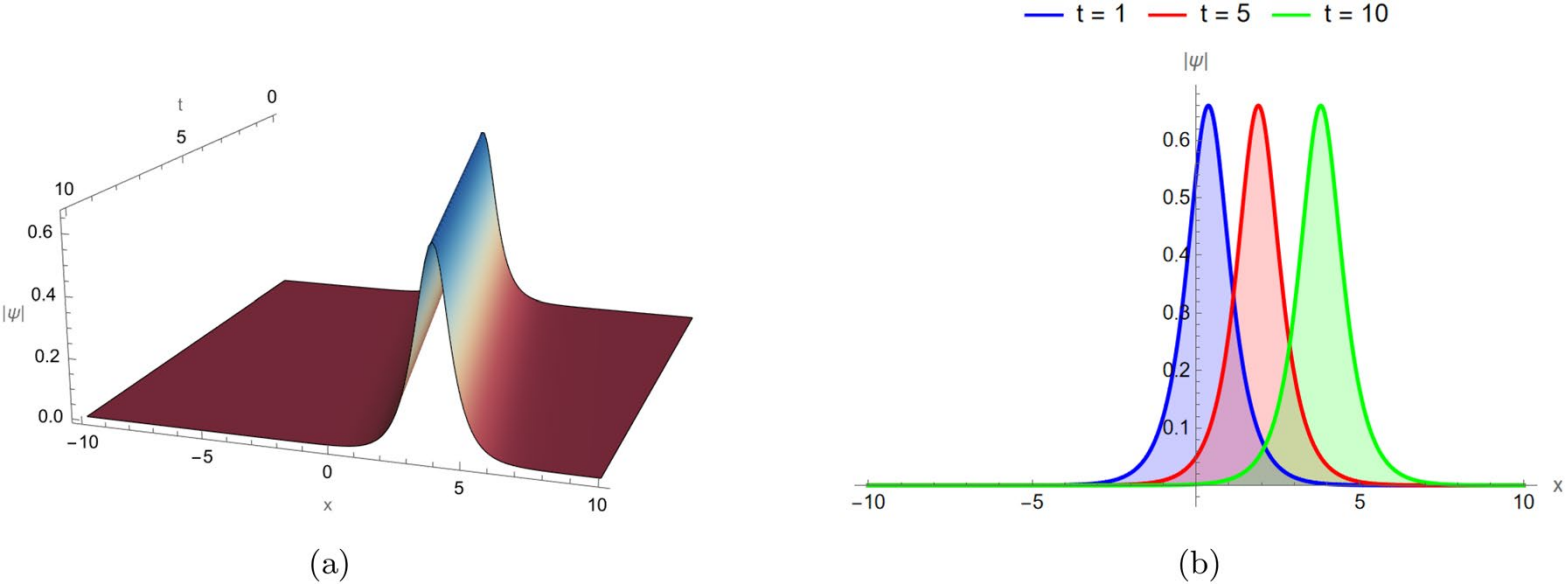
Bright soliton solution of Eq. (8) visualized in two and three dimensions under identical parameter settings: $d_2 = 3$, $\alpha _3 = 5$, $\alpha _4 = 2$, and $\alpha _5 = 1$, and in three different time instances $t=1,5,10$.

Figure 1 shows the bright soliton solution defined by Eq. (8), represented in both two and three dimensions under identical parameter settings: $d_2 = 3$, $\alpha _3 = 5$, $\alpha _4 = 2$, and $\alpha _5 = 1$, and shown at three different snapshots. Bright solitons retain a localized peak as they propagate, owing to the balance between dispersion and nonlinear self-focusing. The soliton maintains its amplitude and width for all plotted time instances $t = 1, 5, 10$, demonstrating stable and localized propagation.

### Dark soliton dynamics

**Fig. 2:**
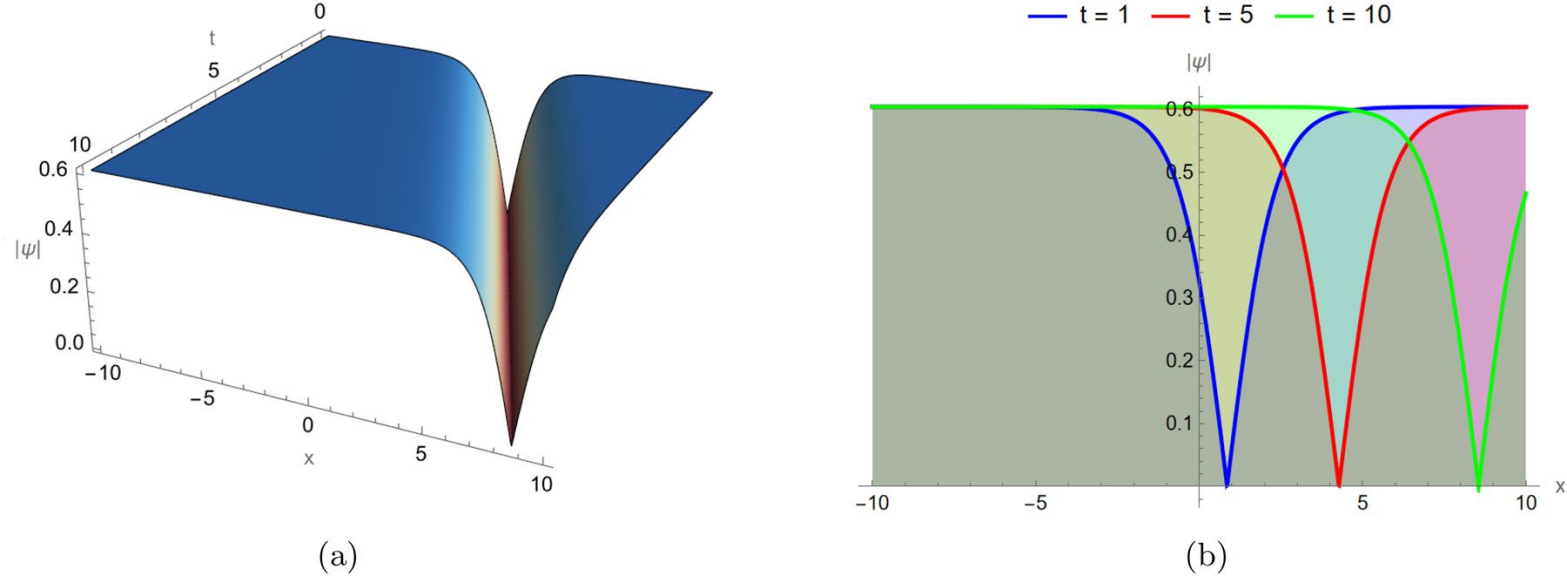
Dark soliton solution for Eq. (9) visualized in two and three dimensions under identical parameter settings: $d_2 = -1$, $\alpha _3 = 3$, $\alpha _4 = 1.5$, and $\alpha _5 = 1$, and in three different time instances $t=1,5,10$.

Figure 2 displays the dark soliton solution governed by Eq. (9), visualized in both two and three dimensions under identical parameter settings: $d_2 = -1$, $\alpha _3 = 3$, $\alpha _4 = 1.5$, and $\alpha _5 = 1$, and in three different time instances. Unlike bright solitons, dark solitons exhibit a localized dip on a continuous background. The structure remains stable for all time levels, reflecting the phase–shifted wave nature observed in defocusing nonlinear media.

### Singular soliton behavior

**Fig. 3:**
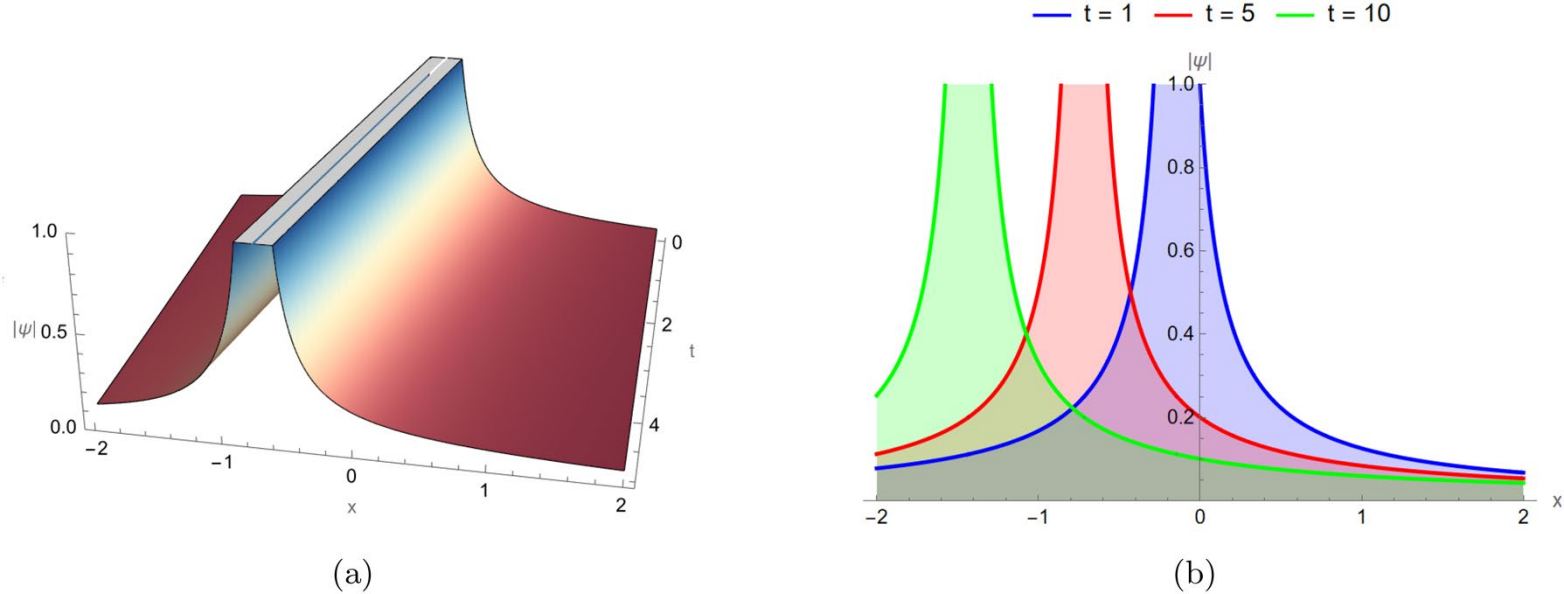
Singular soliton solution for Eq. (14), visualized in two and three dimensions under identical parameter settings: $d_2 = -0.01$, $\alpha _3 = 0.5$, $\alpha _4 = -3$, $\alpha _5 = -1.5$, and in three different time instances $t=1,5,10$.

Figure 3 illustrates the singular soliton solution of Eq. (14), visualized in both two and three dimensions under identical parameter settings: $d_2 = -0.01$, $\alpha _3 = 0.5$, $\alpha _4 = -3$, $\alpha _5 = -1.5$, and in three different time instances. Singular solitons represent a special class of nonlinear wave structures characterized by an infinite or extremely large amplitude at specific spatial points. Such solutions often arise in physical systems where nonlinear effects dominate over dispersion, leading to energy localization and field blow-up. In applications, singular solitons can model intense wave focusing in optical fibers, plasma collapses, or energy concentration in shallow-water dynamics. Singular solitons develop sharp peaks, indicating points where the amplitude becomes extremely large due to dominant nonlinear amplification. The plots show steep localized gradients, highlighting wave concentration phenomena.

### Singular periodic wave profiles

**Fig. 4:**
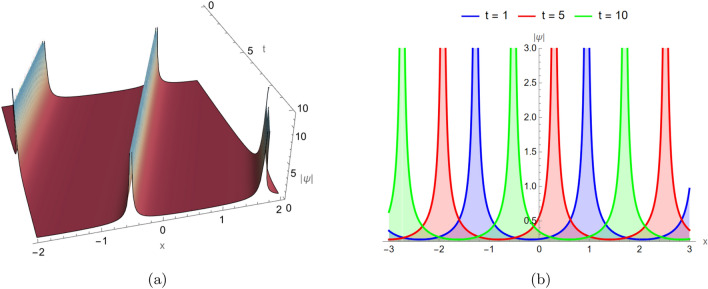
Singular periodic solution for Eq. (17) visualized in two and three dimensions under identical parameter settings: $d_2 = -2$, $\alpha _3 = -5$, $\alpha _4 = -1$, $\alpha _5 = -2.7$, and in three different time instances $t=1,5,10$.

Figure 4 corresponds to the singular periodic solution of Eq. (17), visualized in both two and three dimensions under identical parameter settings: $d_2 = -2$, $\alpha _3 = -5$, $\alpha _4 = -1$, $\alpha _5 = -2.7$, and in three different time instances. These structures periodically develop singular behavior, combining periodic oscillations with sharp amplitude spikes. The plots clearly show repeating localized peaks, indicating strong nonlinear modulation across each period.

### Jacobi elliptic functions and their connection to periodic and soliton solutions

The Jacobi elliptic functions $\textrm{sn}(u,m)$, $\textrm{cn}(u,m)$, and $\textrm{dn}(u,m)$ arise naturally in the analysis of nonlinear evolution equations. Depending on the modulus *m* $(0 \le m \le 1)$, they bridge trigonometric and hyperbolic behaviors, thereby linking periodic and soliton solutions. For $m \rightarrow 0$, they reduce to trigonometric functions:

$$\textrm{sn}(u,0)=\sin (u), \quad \textrm{cn}(u,0)=\cos (u), \quad \textrm{dn}(u,0)=1,$$

while for $m\rightarrow 1$, they become hyperbolic:

$$\textrm{sn}(u,1)=\tanh (u), \quad \textrm{cn}(u,1)=\operatorname {sech}(u), \quad \textrm{dn}(u,1)=\operatorname {sech}(u).$$

**Fig. 5:**
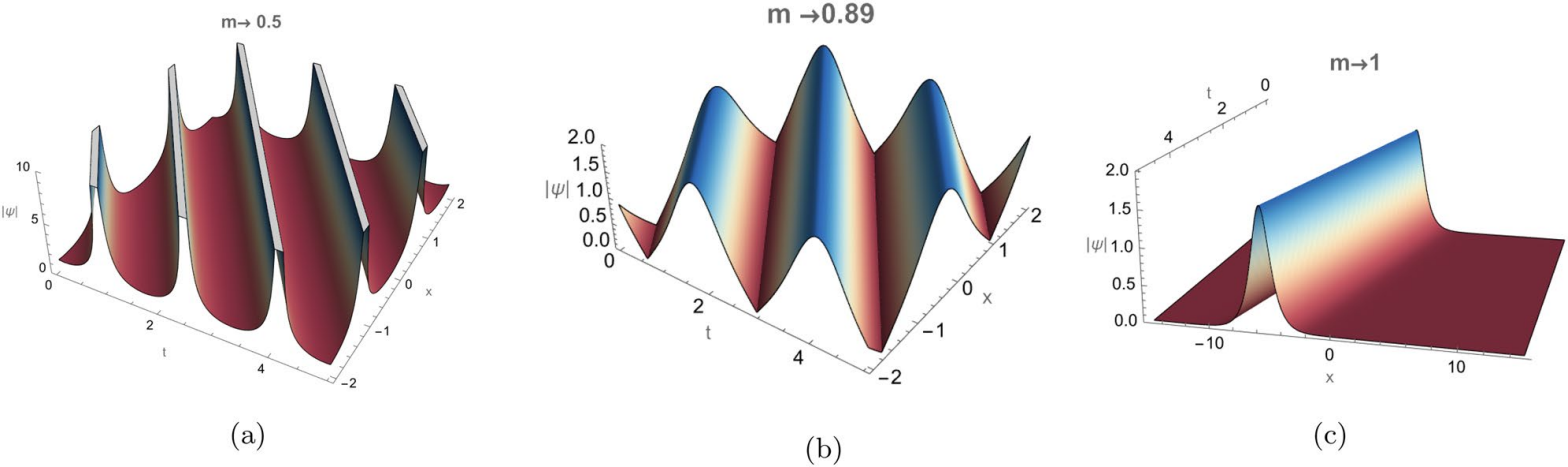
The Jacobi elliptic function $\textrm{cn}(\zeta ,m)$ in Eq. (19) is illustrated in 3D, showing the transition from singular periodic to regular periodic waves and finally to a bright soliton as the modulus $m \rightarrow 1$, with $d_2 = 2$, $\alpha _3 = -3$, $\alpha _4 = 2$, and $\alpha _5 = -1$.

**Fig. 6:**
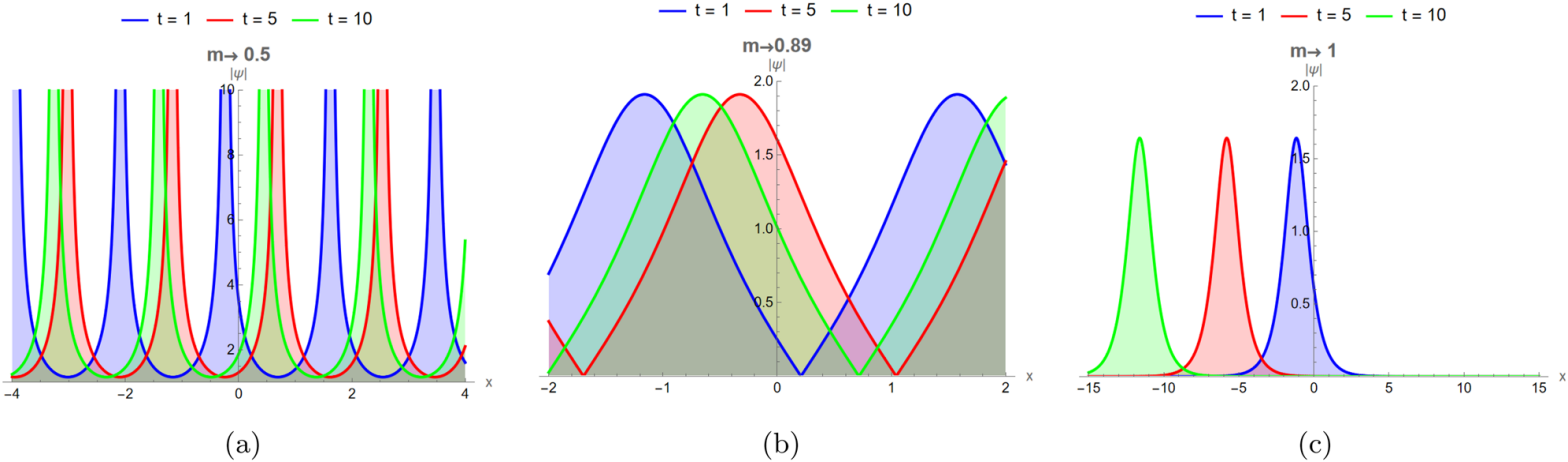
The Jacobi elliptic function $\textrm{cn}(\zeta ,m)$ in Eq. (19) is illustrated in 2D, showing the evolution from singular periodic to regular periodic waves and ultimately to a bright soliton as the modulus $m \rightarrow 1$, with $d_2 = 2$, $\alpha _3 = -3$, $\alpha _4 = 2$, and $\alpha _5 = -1$, at three representative times $t = 1, 5, 10.$.

Thus, varying the modulus *m* from 0 to 1 gradually transforms the periodic Jacobi–elliptic waves into solitary structures. Figure 5 illustrates the evolution of the $\textrm{cn}(\zeta ,m)$ solution in Eq.(19), showing its transition from singular periodic (arising when the argument becomes complex) to regular periodic and finally to bright-soliton profiles in 3D, under the parameter set $d_{2}=2$, $\alpha _{3}=-3$, $\alpha _{4}=2$, and $\alpha _{5}=-1$. Figure 6 presents the corresponding 2D profiles for the same parameter values at three representative times $t=1,5,10$, highlighting the transition from singular periodic to regular periodic and ultimately to bright-soliton behavior.

Overall, the graphical results validate the analytical solutions and demonstrate the complex propagation patterns permitted by the sixth–order NLSE model. The figures highlight the structural evolution, stability characteristics, and nonlinear effects associated with each solution type.

## Conclusion

In this work, we investigated the sixth-order integrable nonlinear Schrödinger equation within its hierarchy and extended the analytical solution landscape of this model. Using the Improved Modified Extended Tanh Function Method (IMETFM), we systematically derived new families of exact solutions, including bright, dark, singular solitons, singular periodic solutions, and Jacobi and Weierstrass elliptic waveforms.

The novelty of this study lies in providing a unified analytical framework for the sixth-order NLSE that captures localized solitons, singular dispersive structures, and doubly-periodic nonlinear waves. The results demonstrate that higher-order dispersion and nonlinear effects significantly enrich the dynamics compared with the classical NLSE, producing additional wave morphologies and amplitude profiles absent in lower-order models.

Two- and three-dimensional graphical simulations illustrate the propagation, intensity localization, and periodic modulation of the solutions, confirming distinct physical signatures, particularly the sharp energy concentration of singular solitons and the smooth periodic patterns of elliptic solutions.

Physically, higher-order dispersive and nonlinear terms enable a broader range of self-trapped and periodic behaviors. Bright and dark solitons correspond to localized energy packets and intensity depressions, while singular solitons represent ultra-localized energy spikes that can model collapse-like events. Jacobi and Weierstrass elliptic solutions bridge localized and periodic states, showing how continuous modulation of the elliptic modulus transitions the system from periodic oscillations to solitary waves, highlighting the delicate balance between dispersion and nonlinearity.

Overall, these findings underline the physical relevance of the sixth-order NLSE hierarchy and the effectiveness of IMETFM for constructing complex analytical waveforms. Future work may explore stability analysis, perturbation dynamics, and parameter sensitivity.

## Data Availability

The datasets used and/or analyzed during the current study are available from the corresponding author upon reasonable request.
